# Higher Global Diet Quality Score Is Inversely Associated with Risk of Type 2 Diabetes in US Women

**DOI:** 10.1093/jn/nxab195

**Published:** 2021-10-23

**Authors:** Teresa T Fung, Yanping Li, Shilpa N Bhupathiraju, Sabri Bromage, Carolina Batis, Michelle D Holmes, Meir Stampfer, Frank B Hu, Megan Deitchler, Walter C Willett

**Affiliations:** Department of Nutrition, Simmons University, Boston, MA, USA; Department of Nutrition, Harvard TH Chan School of Public Health, Boston, MA, USA; Department of Nutrition, Harvard TH Chan School of Public Health, Boston, MA, USA; Department of Nutrition, Harvard TH Chan School of Public Health, Boston, MA, USA; Channing Division of Network Medicine, Department of Medicine, Brigham and Women's Hospital, Harvard Medical School, Boston, MA, USA; Department of Nutrition, Harvard TH Chan School of Public Health, Boston, MA, USA; CONACYT—Health and Nutrition Research Center, National Institute of Public Health, Cuernavaca, Mexico; Channing Division of Network Medicine, Department of Medicine, Brigham and Women's Hospital, Harvard Medical School, Boston, MA, USA; Department of Epidemiology, Harvard TH Chan School of Public Health, Boston, MA, USA; Channing Division of Network Medicine, Department of Medicine, Brigham and Women's Hospital, Harvard Medical School, Boston, MA, USA; Department of Epidemiology, Harvard TH Chan School of Public Health, Boston, MA, USA; Department of Nutrition, Harvard TH Chan School of Public Health, Boston, MA, USA; Channing Division of Network Medicine, Department of Medicine, Brigham and Women's Hospital, Harvard Medical School, Boston, MA, USA; Intake—Center for Dietary Assessment, FHI Solutions, Washington, DC, USA; Department of Nutrition, Harvard TH Chan School of Public Health, Boston, MA, USA; Channing Division of Network Medicine, Department of Medicine, Brigham and Women's Hospital, Harvard Medical School, Boston, MA, USA

**Keywords:** diet quality, diabetes, epidemiology, women, nutrition

## Abstract

**Background:**

We have developed a diet quality metric intended for global use. To assess its utility in high-income settings, an evaluation of its ability to predict chronic disease is needed.

**Objectives:**

We aimed to prospectively examine the ability of the Global Diet Quality Score (GDQS) to predict the risk of type 2 diabetes in the United States, examine potential differences of association by age, and compare the GDQS with other diet quality scores.

**Methods:**

Health, lifestyle, and diet information was collected from women (*n* = 88,520) in the Nurses’ Health Study II aged 27–44 y at baseline through repeated questionnaires between 1991 and 2017. The overall GDQS consists of 25 food groups. Points are awarded for higher intake of healthy groups and lower intake of unhealthy groups (maximum of 49 points). Multivariable HRs were computed for confirmed type 2 diabetes using proportional hazards models. We also compared the GDQS with the Minimum Diet Diversity score for Women (MDD-W) and the Alternate Healthy Eating Index-2010 (AHEI-2010).

**Results:**

We ascertained 6305 incident cases of type 2 diabetes during follow-up. We observed a lower risk of diabetes with higher GDQS; the multivariable HR comparing extreme quintiles of the GDQS was 0.83 (95% CI: 0.76, 0.91; *P*-trend < 0.001). The magnitude of association was similar between women aged <50 y and those aged ≥50 y. An inverse association was observed with lower intake of unhealthy components (HR comparing extreme quintiles of the unhealthy submetric: 0.76; 95% CI: 0.69, 0.84; *P*-trend < 0.001) but was not with the healthy submetric. The inverse association for each 1-SD increase in the GDQS (HR: 0.93; 95% CI: 0.91, 0.96) was stronger (*P* < 0.001) than for the MDD-W (HR: 1.00; 95% CI: 0.94, 1.04) but was slightly weaker (*P* = 0.03) than for the AHEI-2010 (HR: 0.91; 95% CI: 0.88, 0.94).

**Conclusions:**

A higher GDQS was inversely associated with type 2 diabetes risk in US women of reproductive age or older, mainly from lower intake of unhealthy foods. The GDQS performed nearly as well as the AHEI-2010.

## Introduction

Several diet quality indices have been developed and evaluated for their association with risk of chronic diseases (1). These indices typically were based on recommendations for a healthy diet (2–4) or reflections of regional dietary habits (5–7). Many include nutrient components and therefore require the use of a food composition database (2–4), or a scoring algorithm that is based on population-specific intake levels (5, 8). Evidence from prospective studies is consistent that adherence to these diet quality indices is associated with a lower risk of several chronic diseases, including cardiovascular disease and diabetes (1).

To apply these diet quality indices in clinical and public health settings to guide individual dietary choices and public health surveillance, the metric must be simple and quick to administer. In addition, a metric that is valid and practical for use across different parts of the world and different economic development levels would have the additional advantage of enabling global comparisons. Therefore, indices that involve a food composition database or use population-specific scoring would be difficult to implement across regions. To circumvent these limitations, we previously developed the Prime Diet Quality Score (PDQS) that only consists of food groups. It is inversely associated with cardiovascular disease and gestational diabetes in US men and women (9, 10).

To provide a metric that is usable in regions where nutritional adequacy is a concern, we have further modified the PDQS and tested it for association with nutritional markers relevant to middle- and lower-income countries. Our final metric, the Global Diet Quality Score (GDQS), uses a combination of healthy and unhealthy food groups. It has reasonable correlation with measures of nutrient adequacy (11).

Because the GDQS has several differences from the PDQS, we assessed its utility in a higher-income setting by testing its ability to predict the risk of type 2 diabetes in US women. We chose type 2 diabetes because the incidence is increasing globally, with a projected increase from >400 million affected in 2019 to ∼700 million by 2045 (12). In the United States, it was estimated that 12% of adult women and 14% of adult men were living with diabetes in 2013–2016 (13). Although a plethora of medications are available (14), there is no cure in most cases and successful management requires adequate compliance and regular access to health care (15). Therefore, prevention through lifestyle, and especially diet, continues to be an important approach. In this analysis, we prospectively examined the association between the GDQS and the risk of type 2 diabetes among US women, and explored potential differences in association by age. To understand the function of the GDQS, we also explored how the healthy and unhealthy components would drive any observed association. We hypothesized that the overall GDQS and the healthy components (GDQS+ submetric) would be inversely associated with diabetes risk, whereas lower intake of the unhealthy components (GDQS− submetric) would have an inverse association. For the GDQS to be a useful nutrition metric to predict noncommunicable diseases, it must also perform at least similarly as other established diet quality indices. Therefore, we also compared it with the Minimum Diet Diversity score for Women (MDD-W) and the Alternate Healthy Eating Index-2010 (AHEI-2010) for prediction of type 2 diabetes.

## Methods

### Participants

The Nurses’ Health Study II (NHS II) is an ongoing prospective cohort study that is comprised of 116,430 US female Registered Nurses between 25 and 42 y old at inception in 1989 (16). Information on lifestyle practices and incidence of type 2 diabetes was collected every 2 y by self-reported questionnaires. Diet was assessed every 4 y beginning in 1991 using a validated FFQ. Women with diabetes, gestational diabetes, cancer, or cardiovascular disease or who died before the first dietary assessment were excluded. We also excluded those who did not complete additional questionnaires beyond baseline and those who reported implausible energy intakes (<500 or >3500 kcal/d) at baseline. If a participant reported being pregnant in a questionnaire period, person-time during that 2-y period was excluded. A total of 88,520 women were included in this analysis and loss to follow-up was ∼10% during the study period. This study was approved by the institutional review boards of Brigham and Women's Hospital and the Harvard TH Chan School of Public Health.

### Diet assessment

A validated semiquantitative FFQ was self-administered every 4 y, each containing ∼135 items (17). For each food item, a standard portion size was provided with 9 intake frequency choices ranging from “never or less than once per month” to “≥6 times per day.” The GDQS was modified based on the PDQS (9) to capture food groups that would reflect nutrient adequacy and predict major noncommunicable diseases in both lower- and high-income countries globally. It consists of 16 healthy food groups (dark green leafy vegetables, cruciferous vegetables, deep orange vegetables, other vegetables, deep orange fruits, deep orange tubers, citrus fruits, other fruits, legumes, nuts and seeds, poultry and game meats, fish and shellfish, whole grains, liquid oils, low fat dairy, eggs) and 7 unhealthy food groups (white roots and tubers, processed meats, refined grains and baked goods, sugar-sweetened beverages, sweets and ice cream, juices, purchased deep fried foods) (**Supplemental Table 1**). Intake of each food group was classified into <1/wk, 1 to <4/wk, and ≥4/wk. For healthy food groups, points between 0 and 4 were given to each level of intake depending on the food group. For unhealthy food groups, 2, 1, and 0 points were given for the same 3 intake levels so lower intake would receive more points. In addition to the aforementioned food groups, the GDQS also has a red meat group and a full-fat dairy group with different scoring to account for their contribution to nutrient adequacy in low- to middle-income countries. Red meat was given 0, 1, and 0 points for intake of the same 3 intake levels as for the other unhealthy food groups, and full-fat dairy was given 0, 1, 2, and 0 points for intake of <1/wk, 1 to <4/wk, ≥4/wk to <3/d, and ≥3/d, respectively. The full GDQS has 25 food groups and a score range of 0–49 points, with higher points representing a healthier diet. The healthy portion of the GDQS (GDQS+) has a range of 0–32. For the purpose of this analysis, we included red meat and full-fat dairy as part of the unhealthy portion (GDQS−), which has a range of 0–17, with a higher score representing lower intake of unhealthy foods and hence healthier food choices.

To compare the GDQS with other established diet quality indices, we also computed the AHEI-2010 (2) and the MDD-W (18) for each participant. The AHEI-2010 consists of 11 food and nutrient groups. High points are given for higher intakes of healthy groups (vegetables, whole fruits, nuts and legumes, whole grains, polyunsaturated fat, and long-chain n–3 fatty acids) and lower intakes of unhealthy groups (red and processed meats, sugar-sweetened beverages and fruit juice, *trans* fat, and sodium). Points are also given for moderate intake of alcohol. Each component ranges from 0 to 10 points with the total possible score ranging from 0 to 110 points. It has previously been shown to be inversely associated with diabetes risk in women (2).

The MDD-W, originally developed as a proxy indicator for nutrient adequacy, consists of 10 food groups: grains and starchy vegetables, pulses, nuts and seeds, dairy, animal flesh, eggs, dark green leafy vegetables, vitamin A–rich vegetables and fruits, other vegetables, and other fruits (18). The scoring method for the original MDD-W is based on intake collected by 24-h recall. To adapt it for the FFQ, we assigned 1 point for each food group with intake ≥1 serving/d and 0 for less (9). The MDD-W has a range of 0–10 points.

### Outcome assessment

Incident type 2 diabetes was first reported through the biennial questionnaires and confirmed with a validated supplemental questionnaire based on National Diabetes Data Group criteria. This included ≥1 of the following: ≥1 classic symptom (excessive thirst, polyuria or frequent urination, weight loss, hunger), fasting plasma glucose concentrations ≥7.8 mmol/L, or random plasma glucose concentrations ≥11.1 mmol/L (19). In the case of a lack of symptoms, diabetes was considered confirmed with ≥2 elevated plasma glucose concentrations on different occasions (fasting plasma glucose concentrations ≥7.8 mmol/L, random concentrations ≥11.1 mmol/L, and/or 2-h blood glucose concentrations ≥11.1 mmol/L during oral-glucose-tolerance testing); or treatment with hypoglycemic medications (insulin or oral hypoglycemic agent). For cases reported after 1998, criteria from the American Diabetes Association were used in which the threshold for fasting plasma glucose changed from 7.8 mmol/L to 7.0 mmol/L (20). The supplemental questionnaire was validated by a review of medical reports (21). In a random sample of 62 cases in the Nurses’ Health Study that were confirmed by the supplementary questionnaire, 61 (98%) cases were reconfirmed after medical records were reviewed by an endocrinologist blinded to the supplementary questionnaire.

### Covariate assessment

Information on age, race, and height was collected at cohort inception. Body weight, cigarette smoking (including the number of cigarettes per day), physical activity, menopausal status and postmenopausal hormone use, oral contraceptive use, family history of diabetes, history of hypercholesterolemia, and high blood pressure were collected in each biennial questionnaire. BMI (in kg/m^2^) was calculated using height collected at baseline and weight reported at each questionnaire cycle. Alcohol intake and supplemental vitamin and mineral use were collected with FFQs.

### Statistical analysis

For this analysis, follow-up duration in person-years was calculated from the date of return of the 1991 questionnaire to the date of diabetes diagnosis, last questionnaire returned, or 30 June, 2017. We computed cumulative averages of diet quality scores to reduce within-person variation and represent long-term intake (22). We used time-dependent Cox proportional hazards regression models to compute HRs of type 2 diabetes for quintiles of the GDQS, GDQS+, and GDQS−. Eggs are included in the GDQS+ because of their protein and vitamin content, but they also contain substantial amounts of cholesterol. Hence, we in addition computed an alternate GDQS+ without the egg component for sensitivity analysis. We tested for the proportional hazards assumption by including an interaction term of GDQS and age (which reflects time) and used the likelihood ratio test. The *P* value for the chi-square distribution was >0.05, hence it did not show a violation of the proportional hazards assumption.

All models were adjusted by age (mo) at the start of follow-up for each woman and the calendar year of each questionnaire cycle. Multivariable models were adjusted for race (white/nonwhite), family history of diabetes, smoking (never, past, 1–14 cigarettes/d, 15–24 cigarettes/d, ≥25 cigarettes/d), alcohol intake (none, <5 g/d, 5 to <10 g/d, ≥10 g/d), energy intake (quintiles), coffee intake (continuous), physical activity [<3 metabolic equivalent hours (METs)/wk, 3 to <9 METs/wk, 9 to <18 METs/wk, 18 to <27 METs/wk, ≥27 METs/wk], BMI (<23, 23 to < 25, 25 to <30, 30 to <35, ≥35), multivitamin use (yes/no), menopausal status and menopausal hormone therapy (premenopausal, no hormone use, past use, current use), oral contraceptive use (never, past, current), history of hypertension at baseline, and history of hyperlipidemia at baseline. We used restricted cubic spline regression to assess potential nonlinear association. To access potential differential association of the GDQS with diabetes by age, we conducted analyses stratified by age. We also stratified the analysis by BMI status and physical activity. To examine the potential influence of pregnancy on the association between the GDQS and diabetes, we ran regression models separately for women based on pregnancy history, and among ever-pregnant women by history of gestational diabetes. Tests for 2-way interaction between the GDQS and each of the stratified factors were conducted using the likelihood ratio test comparing regression models with and without an interaction term. Analysis was conducted using SAS version 9.4 (SAS Institute Inc.).

To compare the strength of association between the GDQS and the AHEI-2010 and MDD-W, we standardized each score and modeled each 1 SD of the scores in the same model. Differences in the regression coefficients were compared using the Wald test.

## Results

In ≤26 y of follow-up, we ascertained 6305 incident cases of type 2 diabetes, of which 2266 were women younger than 50 y old and 4039 were women ≥50 y old. Women with a higher GDQS tended to be leaner, more physically active, less likely to be current smokers, and consumed more alcohol and coffee (**Table 1**).

**TABLE 1:** Age-standardized baseline characteristics by quintiles of GDQS in the Nurses’ Health Study II^1^

	Q1	Q2	Q3	Q4	Q5
BMI	24.8 ± 5.8	24.6 ± 5.5	24.4 ± 5.1	24.3 ± 5.0	24.2 ± 4.8
Physical activity, METs	14.5 ± 21.2	17.8 ± 24.1	20.4 ± 26.2	23.5 ± 28.7	29.1 ± 34.0
Current smoker, %	18	14	12	11	9
GDQS	14.3 ± 2.2	18.7 ± 0.9	21.5 ± 0.8	24.4 ± 0.9	28.8 ± 2.2
Unhealthy GDQS components	7.1 ± 2.3	8.2 ± 2.4	8.6 ± 2.4	9.1 ± 2.4	10.1 ± 2.2
Healthy GDQS components	7.3 ± 2.8	10.7 ± 2.5	12.9 ± 2.5	15.3 ± 2.4	18.7 ± 2.7
MDD-W	3.0 ± 1.3	3.6 ± 1.3	4.1 ± 1.3	4.6 ± 1.2	5.4 ± 1.2
AHEI-2010	37.8 ± 7.6	43.7 ± 7.7	47.8 ± 7.9	52.0 ± 8.3	58.8 ± 8.8
Energy intake, kcal/d	1641 ± 536	1689 ± 537	1743 ± 532	1831 ± 530	1990 ± 529
Fiber, g/d	14.3 ± 3.6	16.5 ± 4.0	18.2 ± 4.8	20.0 ± 5.2	22.7 ± 5.8
Alcohol, g/d	2.4 ± 5.7	3.0 ± 6.3	3.3 ± 6.2	3.5 ± 6.1	3.9 ± 6.5
Processed meats, servings/d	0.31 ± 0.33	0.26 ± 0.28	0.22 ± 0.25	0.19 ± 0.23	0.15 ± 0.20
Red meats, servings/d	0.67 ± 0.43	0.60 ± 0.41	0.55 ± 0.38	0.52 ± 0.37	0.44 ± 0.34
Vegetables, servings/d	1.8 ± 1.0	2.5 ± 1.3	3.0 ± 1.5	3.8 ± 1.7	5.1 ± 2.4
Fruit, servings/d	1.2 ± 1.0	1.5 ± 1.1	1.8 ± 1.2	2.1 ± 1.3	2.6 ± 1.6
Nuts and seeds, servings/d	0.04 ± 0.08	0.05 ± 0.10	0.06 ± 0.11	0.07 ± 0.16	0.11 ± 0.21
Legumes, servings/d	0.16 ± 0.16	0.20 ± 0.18	0.24 ± 0.23	0.29 ± 0.26	0.41 ± 0.35
Coffee, servings/d	1.4 ± 1.7	1.5 ± 1.7	1.6 ± 1.7	1.7 ± 1.7	1.8 ± 1.7

^1^ *n* = 88,520. Values are means ± SDs unless otherwise indicated. AHEI-2010, Alternate Healthy Eating Index-2010; GDQS, Global Diet Quality Score; MDD-W, Minimum Diet Diversity score for Women; MET, metabolic equivalent hour; Q, quintile.

We observed a lower risk of diabetes with higher GDQS (multivariable HR comparing extreme quintiles: 0.83; 95% CI: 0.76, 0.91; *P*-trend < 0.001) (**Table 2**). The association for women age <50 y was 0.85 (95% CI: 0.73, 0.98; *P*-trend < 0.001) and for age ≥50 y was 0.82 (95% CI: 0.74, 0.91, *P*-trend < 0.001) with no significant interaction. We also separately examined the submetrics of the GDQS representing healthy (GDQS+) and unhealthy (GDQS−) food components. These 2 submetrics were only weakly correlated (Spearman *r* = −0.06, *P* < 0.001). The healthy components of the GDQS (GDQS+) were not associated with diabetes risk (**Table 3**). On the other hand, higher GDQS−, which represents lower intake of the unhealthy components, showed an inverse association (multivariable HR comparing extreme quintiles: 0.76; 95% CI: 0.69, 0.84; *P*-trend < 0.001). There was no apparent difference in association by age. Spline regression did not detect significant departure from linearity for the overall GDQS, GDQS+, or GDQS− (data not shown). In the sensitivity analysis in which we excluded the egg component from the GDQS+, the null association persisted in the remaining portion of the GDQS+.

**TABLE 2:** HRs (95% CI) for type 2 diabetes according to quintiles of the Global Diet Quality Score in the Nurses’ Health Study II^1^

	Q1	Q2	Q3	Q4	Q5	*P*-trend
All women						
Median score	15.8	19.5	21.9	24.4	27.8	
Cases, *n*	1647	1309	1262	1112	975	
Person-years	365,779	364,667	365,382	373,363	364,174	
Age- and kcal-adjusted	1	0.76 (0.71, 0.82)	0.71 (0.66, 0.76)	0.59 (0.55, 0.64)	0.48 (0.44, 0.52)	<0.001
Multivariable^2^	1	0.91 (0.84, 0.97)	0.94 (0.87, 1.01)	0.87 (0.80, 0.94)	0.83 (0.76, 0.91)	<0.001
Women < age 50 y						
Median score	15.3	18.9	21.3	23.8	27.3	
Cases, *n*	634	456	459	395	322	
Person-years	210,566	202,881	198,185	201,222	184,898	
Age- and kcal-adjusted	1	0.72 (0.64, 0.82)	0.73 (0.65, 0.83)	0.61 (0.53, 0.69)	0.50 (0.44, 0.58)	<0.001
Multivariable^2^	1	0.86 (0.76, 0.98)	1.00 (0.88, 1.13)	0.90 (0.79, 1.02)	0.85 (0.73, 0.98)	0.02
Women age ≥ 50 y						
Median score	16.7	20.3	22.8	25.0	28.1	
Cases, *n*	1013	853	803	717	653	
Person-years	155,214	161,786	167,196	172,140	179,276	
Age- and kcal-adjusted	1	0.79 (0.72, 0.86)	0.70 (0.63, 0.76)	0.58 (0.53, 0.64)	0.47 (0.43, 0.52)	<0.001
Multivariable^2^	1	0.93 (0.85, 1.02)	0.91 (0.82, 1.00)	0.85 (0.77, 0.94)	0.82 (0.74, 0.91)	<0.001

1 *n* = 88,520. Q, quintile.

2 Adjusted for age, BMI, energy intake, smoking, family history of diabetes, oral contraceptive use, menopausal status and postmenopausal hormone use (“all women” analysis only), physical activity, alcohol intake, and multivitamin use.

**TABLE 3:** HRs (95% CI) for type 2 diabetes according to quintiles of the healthy (GDQS+) and unhealthy (GDQS−) submetrics of the GDQS in the Nurses’ Health Study II^1^

	Q1	Q2	Q3	Q4	Q5	*P*-trend
GDQS+ submetric (max = 32)						
All women						
Median score	8.0	11.3	13.6	15.8	18.8	
Cases, *n*	1441	1290	1188	1232	1154	
Person-years	366,057	365,828	368,066	366,408	367,005	
Age- and kcal-adjusted	1	0.83 (0.77, 0.90)	0.70 (0.64, 0.76)	0.67 (0.62, 0.73)	0.54 (0.49, 0.59)	<0.001
Multivariable^2^	1	1.00 (0.92, 1.08)	0.98 (0.90, 1.07)	1.05 (0.96, 1.14)	1.00 (0.91, 1.10)	0.86
Women < age 50 y						
Median score	7.5	10.8	13.2	15.4	18.5	
Cases, *n*	554	459	403	443	407	
Person-years	205,773	202,984	200,220	197,583	191,192	
Age- and kcal-adjusted	1	0.79 (0.69, 0.89)	0.64 (0.56, 0.73)	0.67 (0.58, 0.76)	0.55 (0.47, 0.64)	<0.001
Multivariable^2^	1	0.96 (0.84, 1.09)	0.92 (0.80, 1.06)	1.04 (0.90, 1.20)	1.00 (0.85, 1.17)	0.97
Women age ≥ 50 y						
Median score	8.6	11.9	14.1	16.2	19.1	
Cases, *n*	887	831	785	789	747	
Person-years	160,284	162,845	167,846	168,825	175,813	
Age- and kcal-adjusted	1	0.86 (0.78, 0.94)	0.74 (0.67, 0.82)	0.68 (0.61, 0.76)	0.54 (0.48, 0.60)	<0.001
Multivariable^2^	1	1.02 (0.92, 1.13)	1.03 (0.92, 1.14)	1.05 (0.94, 1.18)	1.01 (0.89, 1.14)	0.77
GDQS− submetric (max = 14) (high score = less unhealthy)						
All women						
Median score	5.5	7.2	8.5	9.6	11.0	
Cases, *n*	1701	1446	1151	1050	957	
Person-years	374,851	354,527	367,116	359,807	377,063	
Age- and kcal-adjusted	1	0.84 (0.78, 0.90)	0.66 (0.61, 0.71)	0.56 (0.51, 0.61)	0.47 (0.43, 0.51)	<0.001
Multivariable^2^	1	0.96 (0.89, 1.04)	0.85 (0.78, 0.92)	0.80 (0.73, 0.88)	0.76 (0.69, 0.84)	<0.001
Women < age 50 y						
Median score	5.3	7.0	8.0	9.5	11.0	
Cases, *n*	661	517	394	375	319	
Person-years	219,316	190,611	202,692	190,611	194,521	
Age- and kcal-adjusted	1	0.86 (0.76, 0.97)	0.69 (0.60, 0.79)	0.61 (0.53, 0.70)	0.52 (0.44, 0.61)	<0.001
Multivariable^2^	1	0.96 (0.85, 1.09)	0.87 (0.76, 1.00)	0.83 (0.72, 0.97)	0.81 (0.68, 0.95)	<0.001
Women age ≥ 50 y						
Median score	5.8	7.5	8.7	9.8	11.2	
Cases, *n*	1040	929	757	675	638	
Person-years	155,535	163,915	164,424	169,196	182,542	
Age- and kcal-adjusted	1	0.82 (0.75, 0.90)	0.64 (0.58, 0.71)	0.54 (0.48, 0.60)	0.45 (0.40, 0.50)	<0.001
Multivariable^2^	1	0.96 (0.87, 1.05)	0.83 (0.75, 0.92)	0.78 (0.70, 0.88)	0.74 (0.65, 0.83)	<0.001

1 *n* = 88,520. GDQS, Global Diet Quality Score; Q, quintile.

2 Adjusted for age, BMI, energy intake, smoking, family history of diabetes, oral contraceptive use, menopausal status and postmenopausal hormone use (“all women” analysis only), physical activity, alcohol intake, multivitamin use, and mutually adjusted for the other submetric.

The GDQS was inversely associated with diabetes in both women ever or never pregnant (**Supplemental Table 2**). Although the magnitude of association did not differ substantially for pregnancy history, the trend appeared to be more consistent for never-pregnant women (*P*-interaction = 0.06). Among women who had been pregnant, an inverse association with the GDQS was only observed for those without a history of gestational diabetes (multivariable HR comparing extreme quintiles: 0.83; 95% CI: 0.75, 0.91; *P*-trend < 0.001). We also stratified the analysis by BMI and physical activity (**Supplemental Table 3**). The inverse association was significant regardless of BMI status; however, it was stronger among leaner women (*P*-interaction < 0.001). On the other hand, although the association between the GDQS and diabetes appeared stronger among those with physical activity above the median, the *P* value for interaction did not reach statistical significance.

**FIGURE 1:**
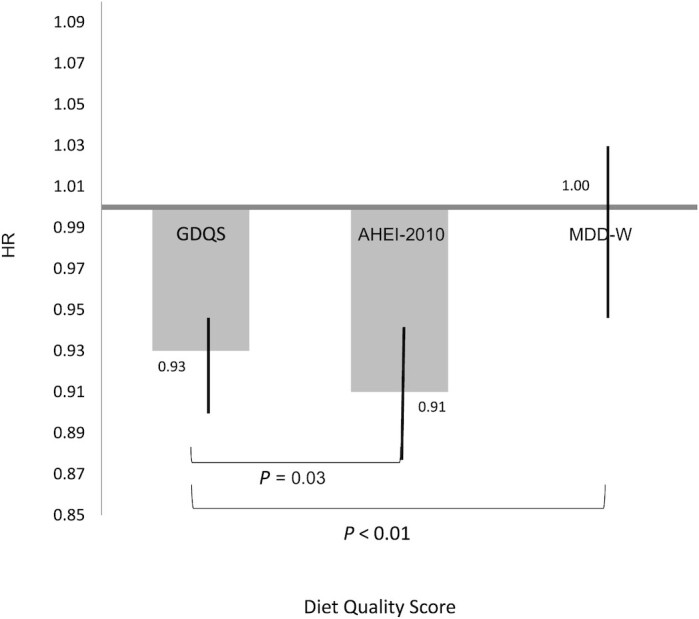
Multivariable HR for a 1-SD increase of the GDQS, AHEI-2010, and MDD-W. Models were adjusted for age, BMI, energy intake, smoking, family history of diabetes, oral contraceptive use, menopausal status and postmenopausal hormone use (“all women” analysis only), physical activity, alcohol intake, and multivitamin use. Vertical lines represent 95% CIs. Chi-square test *P* values tested for significant differences in HR between the GDQS and AHEI-2010, and GDQS and MDD-W. AHEI-2010, Alternate Healthy Eating Index-2010; GDQS, Global Diet Quality Score; MDD-W, Minimum Diet Diversity score for Women.

We also compared the magnitude of association of the GDQS with 2 other diet quality scores: the AHEI-2010 and MDD-W. The Spearman correlation coefficient between the GDQS and the AHEI-2010 was 0.74 (*P* < 0.001); it was 0.64 (*P* < 0.001) with the MDD-W. The AHEI-2010 was inversely associated with diabetes (multivariable HR comparing extreme quintiles: 0.62; 95% CI: 0.56, 0.68; *P*-trend < 0.001) and there was no appreciable difference by age (**Supplemental Table 4**). However, no association was observed with the MDD-W (**Supplemental Table 5**). When we compared the association of the GDQS with diabetes pairwise with the AHEI-2010 and the MDD-W, the association for each SD increase in the AHEI-2010 was slightly stronger than for the GDQS (HR: 0.91 compared with 0.93, *P* for difference = 0.03) (**Figure 1**). On the other hand, the association for the GDQS was clearly stronger than for the MDD-W (*P* for difference < 0.001).

## Discussion

In this analysis, we observed an inverse association between a diet quality score designed for global use and risk of type 2 diabetes among US women. The association appeared to be driven by lower intakes of unhealthy foods. The GDQS compared well with the AHEI-2010 which showed a strong inverse association with diabetes in a cohort of middle-aged nurses (23). The lower diabetes risk with a higher GDQS was similar between women of reproductive age and those who were older.

Prospective studies from the United States (24), Europe (6), and Asia (25, 26) have shown adherence to healthy eating guidelines, as reflected by higher diet quality indices, to be associated with lower risk of type 2 diabetes. Although different diet quality indices were used in these studies, such as the Healthy Diet Score, the Healthy Eating Index, the Alternate Healthy Eating Index, and some form of Mediterranean diet score, the common features among them were higher intakes of fruits, vegetables, whole grains, and lean protein and lower intakes of red and processed meats, added sugar, and refined grains. The number of components ranged from 6 in the Healthy Nordic Food Index (6) to 11 in the Alternate Healthy Eating Index (24). The GDQS features similar food groups, but in more refined categories and hence a total of 25 food groups. We have chosen the approach of using more specific food groups to better specify nutrients, such as vitamin C and provitamin A carotenoids that are nutrients of concern in some parts of the world.

In our analysis, lower intakes of foods in the unhealthy submetric of the GDQS (GDQS−) were more strongly associated with a lower diabetes risk than was the healthy submetric of the GDQS (GDQS+). Among the foods in the GDQS−, high intakes of red and processed meats (27), refined grains (28), sugar-sweetened beverages (28), and potatoes, especially as French fries (29), have previously been shown to be directly associated with higher risk of type 2 diabetes. In addition, fried foods have also been shown to increase risk of type 2 diabetes (30) or gestational diabetes (31) in US women. Fried foods may be a risk factor for diabetes owing to the high energy content or the increase in lipid oxidation products (32) and *trans* fat (33) created in the process of frying. Red and processed meat may be involved in the pathogenesis of type 2 diabetes through inducing proinflammatory advanced glycation end products (34) and pancreatic injury due to oxidative stress from heme iron (35). In addition, nitrites and nitrates in processed meats could be precursors for the pro-oxidant peroxynitrate (36). Refined grains and sugar-sweetened beverages may contribute to weight gain (37) and the high glycemic load has been associated with diabetes risk (38).

Healthy dietary patterns similar to the healthy submetric of the GDQS (GDQS+) are inversely associated with diabetes (39). However, a meta-analysis only found marginally significant inverse associations for individual food groups such as fruits, vegetables, and nuts (28). Our analysis also did not observe an inverse association of the GDQS+ with diabetes, even when the egg component, which has been associated with diabetes risk in US studies (40), was removed. Although the GDQS+ encompasses a number of healthy food groups and can potentially detect joint association of these food groups, each food group only has 3 levels of scoring. It is possible that only high intakes of specific foods or food groups are associated with lower risk of diabetes and our scoring could not differentiate these high intakes. On the other hand, the food groups in the unhealthy submetric might be more strongly associated with diabetes than our scoring method was sufficient to detect.

The strengths of this study include the large sample size and long follow-up which allowed us to accrue a sufficient number of cases to examine diabetes risk even among women of reproductive age. The detailed and repeated assessment of lifestyle and health information in the Nurses’ Health Study II allowed us to explore potential difference in risk by reproductive history. On the other hand, lifestyle and diet information was obtained from self-report. Although the validity of the dietary questionnaire has been well documented (41), some degree of misclassification is inevitable. And although we have adjusted for multiple confounders that were updated throughout follow-up, we cannot exclude the possibility of residual confounding.

In designing the GDQS, the metric has to be applicable to geographical regions with a wide range of economic resources and nutrition challenges. Therefore, the score was constructed to balance the needs to reflect nutrient adequacy and predict chronic disease risk. For that purpose, the red meat component which would normally be considered as unhealthy in high-income countries was given 1 point for moderate intake and 0 for low or high intake, to recognize its value as a protein and iron source in lower-resource regions. Similarly, points were given for moderate consumption of full-fat dairy to recognize its value as a protein, calcium, and energy source, but we did not award points for very high or no consumption. Also, the GDQS promotes moderate consumption of poultry, fish, eggs, and low fat dairy.

Because the GDQS was not designed specifically to predict the risk of diabetes, it does not include coffee (42) and moderate alcohol consumption in the metric score (43), both of which are inversely associated with type 2 diabetes risk. Nevertheless, we were still able to observe a strong association with type 2 diabetes risk, and the GDQS performed well against 2 other diet quality scores. In particular, the GDQS is easier to use than the AHEI-2010. The GDQS, however, reflects overall diet healthfulness and is not specifically aimed for the prevention of a specific disease. As a result, a high GDQS does not represent the optimal dietary characteristics for the prevention of diabetes.

In the current global drive to shift food consumption to be more plant focused for both human and planetary health (44), the food groups chosen for the GDQS have implicit concordance with this goal. Out of the 17 healthy food groups to emphasize in the diet, only 4 were from animal origin. And out of the 9 unhealthy food groups to minimize intake, 3 were animal protein, and 1 (sweets and ice cream) often has ingredients from animal origin. Therefore, a diet that scores high on the GDQS would tend to be correlated with diets that are relatively more plant-based.

Health metrics that have specific cutoffs are useful for risk assessment and setting treatment targets. Clinically relevant cutoffs can be identified if there are inflection points in the relation of the GDQS and risk of diabetes. Cutoffs can also be set by assigning a priori categories. However, this latter approach requires somewhat arbitrary decisions and also needs to consider other outcomes and diverse populations. In our results, there was no departure from linearity in the GDQS. Because our results point toward a progressively lower risk of diabetes with higher GDQS, there is no strong premise to support specific cutoffs for the GDQS in this cohort of US women.

In conclusion, the GDQS was inversely associated with type 2 diabetes in both reproductive-age and older women in a high-income country. It performed well compared with the AHEI-2010 in predicting diabetes risk and our results showed that lower intake of unhealthy foods appeared to be more important than higher intake of healthy foods. Further testing of the GDQS in other populations is needed to confirm its usefulness in a broad range of populations to predict noncommunicable diseases.

## Supplementary Material

nxab195_Supplemental_File

## Notes

Funding for the research was provided by FHI Solutions, recipient of a Bill & Melinda Gates Foundation grant to support Intake—Center for Dietary Assessment and by NIH grant U01 CA176726.

Author disclosures: TTF is an Associate Editor for the Journal of Nutrition and played no role in the Journal’s evaluation of the manuscript. All other authors report no conflicts of interest.

Supplemental Tables 1–5 are available from the “Supplementary data” link in the online posting of the article and from the same link in the online table of contents at https://academic.oup.com/jn.

Published in a supplement to *The Journal of Nutrition*. Publication costs for this supplement were funded by the Bill & Melinda Gates Foundation in conjunction with FHI Solutions, recipient of a Bill & Melinda Gates Foundation grant to support Intake—Center for Dietary Assessment. The opinions expressed in this publication are those of the authors and are not attributable to the sponsors or the publisher, Editor, or Editorial Board of *The Journal of Nutrition*. The Supplement Coordinators for the supplement publication were Megan Deitchler, Intake—Center for Dietary Assessment at FHI Solutions, Washington, DC; and Sabri Bromage, Harvard TH Chan School of Public Health, Boston, MA.

The GDQS research initiative was launched by Intake – Center for Dietary Assessment. The research was led by Harvard T.H. Chan School of Public Health, Department of Nutrition and carried out in collaboration with researchers at the National Public Health Institute (INSP), Mexico. Funding for the research was provided by FHI Solutions, recipient of a Bill & Melinda Gates Foundation grant to support Intake – Center for Dietary Assessment.

Abbreviations used: AHEI-2010, Alternate Healthy Eating Index-2010; GDQS, Global Diet Quality Score; MDD-W, Minimum Diet Diversity score for Women; MET, metabolic equivalent hour; PDQS, Prime Diet Quality Score.
