# Supplementary material for: Higher Global Diet Quality Score Is Inversely Associated with Risk of Type 2 Diabetes in US Women
Source: J Nutr. 2021 Oct 23;151(Suppl 2):168S–175S. doi: 10.1093/jn/nxab195 (PMC8542093; doi:10.1093/jn/nxab195)
Supplement: nxab195_Supplemental_File [file nxab195_supplemental_file.pdf]

Online Supplementary Material

Supplemental table 1: Scoring criteria (number of points for each intake level) for the Global Diet Quality Score

Food group	0 to <1/week	1/week to < 4/week	≥4/week
**Healthy food groups**			
Dark green leafy vegetables	0	2	4
Cruciferous vegetables	0	0.25	0.5
Deep orange vegetables	0	0.25	0.5
Deep orange fruits	0	1	2
Deep orange tubers	0	0.25	0.5
Citrus fruits	0	1	2
Other vegetables	0	0.25	0.5
Other fruits	0	1	2
Legumes	0	2	4
Nuts and seeds	0	1	2
Poultry	0	1	2
Fish	0	1	2
Whole grains	0	1	2
Liquid oils	0	1	2
Low fat dairy	0	1	2
Eggs	0	1	2
**Unhealthy food groups**			
White roots and tubers	2	1	0
Process meats	2	1	0
Refined grains and baked goods	2	1	0
Sugar sweetened beverages	2	1	0
Sweets and ice-cream	2	1	0
Juices	2	1	0
Fried foods	2	1	0
Red meats	0	1	0
Full fat dairy[1]	0	1, 2	0

1. 1 point for 1/week to < 4/week, 2 points for 4/week to < 3/day, 0 point for ≥3/day

Supplemental table 2: Multivariable[1] hazard ratio for type 2 diabetes by quintiles of Global Diet Quality Score stratified by pregnancy history and history of gestational diabetes (GDM) in the Nurses' Health Study II (*n*=88,520)

	Q1	Q2	Q3	Q4	Q5	*P* trend
**Pregnancy history**[2]						
Never pregnant (case=1164)	1	0.94 (0.78, 1.13)	0.88 (0.73,1.06)	0.86 (0.71, 1.05)	0.79 (0.65,0.98)	0.005
Ever pregnant (case=5155)	1	0.89 (0.82, 0.97)	0.94 (0.86, 1.02)	0.87 (0.79, 0.95)	0.83 (0.75, 0.91)	<0.001
**Ever pregnant women only**[3]						
No history of GDM (case=5079)	1	0.94 (0.86,1.02)	0.95 (0.87,1.03)	0.90 (0.83,0.99)	0.83 (0.76,0.92)	<0.001
History of GDM (case=933)	1	0.81 (0.64,1.02)	1.02 (0.81, 1.27)	0.87 (0.69,1.10)	1.02 (0.82, 1.27)	0.56

1. Adjusted for age, BMI, energy intake, smoking, family history of diabetes, oral contraceptive use, menopausal status and postmenopausal hormone use ("all women" analysis only), physical activity, alcohol intake, multivitamin use.
2. p for interaction = 0.06
3. p for interaction = 0.34

Supplemental table 3: Multivariable[1] hazard ratio for type 2 diabetes by quintiles of Gobal Diet Quality Score stratified by BMI and physical activity in the Nurses' Health Study II (n=88,520)

	Q1	Q2	Q3	Q4	Q5	*P* trend	*P* interaction
**All women**							
BMI< 25.0 (case= 347)	1	0.77 (0.56, 1.06)	0.67 (0.48, 0.93)	0.58 (0.40, 0.82)	0.65 (0.46, 0.93)	0.006	
BMI ≥ 25.0 (case=5743)	1	0.92 (0.85, 0.99)	0.96 (0.89, 1.04)	0.89 (0.82, 0.97)	0.85 (0.77, 0.93)	<0.001	0.04
Physical activity < median (case= 4329)	1	0.92 (0.85, 1.01)	0.92 (0.84, 1.01)	0.85 (0.77, 0.94)	0.87 (0.79, 0.97)	0.002	
Physical activity ≥ median (case= 1990)	1	0.83 (0.71, 0.96)	0.90 (0.77, 1.04)	0.83 (0.71, 0.96)	0.73 (0.62, 0.85)	<0.001	0.96
**Women < age 50**							
BMI< 25.0 (case= 129)	1	0.62 (0.37, 1.02)	0.51 (0.29, 0.88)	0.46 (0.26, 0.84)	0.64 (0.35, 1.14)	0.03	
BMI ≥ 25.0 (case= 2061)	1	0.89 (0.78, 1.01)	1.05 (0.92, 1.20)	0.92 (0.80. 1.06)	0.87 (0.75, 1.02)	0.10	<0.001
Physical activity < median (case= 1507)	1	0.85 (0.74, 0.99)	0.96 (0.83, 1.01)	0.84 (0.71, 0.99)	0.91 (0.76, 1.10)	0.08	
Physical activity ≥ median (case= 762)	1	0.88 (0.69, 1.12)	1.04 (0.82, 1.32)	0.95 (0.75, 1.21)	0.78 (0.60, 1.00)	0.08	<0.44
**Women age 50+**							
BMI< 25.0 (case= 218)	1	0.90 (0.60, 1.36)	0.81 (0.52, 1.24)	0.67 (0.43, 1.06)	0.70 (0.44, 1.11)	0.11	
BMI ≥ 25.0 (case= 3682)	1	0.93 (0.84, 1.03)	0.92 (0.83, 1.01)	0.88 (0.79, 0.97)	0.83 (0.74, 0.93)	0.001	<0.001
Physical activity < median (case= 2822)	1	0.96 (0.87, 1.07)	0.91 (0.81, 1.02)	0.86 (0.76, 0.97)	0.85 (0.75, 0.97)	0.01	
Physical activity ≥ median (case= 1228)	1	0.78 (0.64, 0.95)	0.81 (0.67, 0.98)	0.75 (0.62, 0.90)	0.68 (0.56, 0.82)	<0.001	0.72

1. Adjusted for age, BMI, energy intake, smoking, family history of diabetes, oral contraceptive use, menopausal status and postmenopausal hormone use ("all women" analysis only), physical activity, alcohol intake, multivitamin use

Supplemental table 4: Hazard ratio for type 2 diabetes according to quintiles of Alternate Healthy Eating Index-2010 in the Nurses' Health Study II (*n*=88,520)

	**Q1**	**Q2**	**Q3**	**Q4**	**Q5**	***P* trend**
**All women**						
Median score	37.9	45.0	50.2	56.0	64.9	
No. of cases	1812	1474	1261	1049	709	
Person years	364,689	366,529	366,920	367,391	367,835	
Age &kcal adjusted	1	0.80 (0.74, 0.85)	0.66 (0.61, 0.71)	0.53 (0.49, 0.58)	0.35 (0.32, 0.38)	<0.001
Multivariable[1]	1	0.95 (0.89, 1.02)	0.88 (0.82, 0.95)	0.80 (0.74, 0.87)	0.62 (0.56, 0.68)	<0.001
**Women < age 50**						
Median score	36.5	43.5	48.7	54.2	62.5	
No. of cases	739	535	447	322	223	
Person years	214,332	207,304	199,858	193,004	183,253	
Age &kcal adjusted	1	0.75 (0.67, 0.84)	0.65 (0.58, 0.73)	0.48 (0.42, 0.55)	0.35 (0.30, 0.41)	<0.001
Multivariable[1]	1	0.91 (0.81, 1.02)	0.89 (0.78, 1.00)	0.76 (0.66, 0.88)	0.64 (0.55, 0.75)	<0.001
**Women age ≥50**						
Median score	40.1	47.5	52.8	58.3	66.7	
No. of cases	1073	939	814	727	486	
Person years	150,356	159,225	167,062	174,387	184,582	
Age &kcal adjusted	1	0.83 (0.76, 0.90)	0.67 (0.61 0.73)	0.56 (0.51, 0.62)	0.35 (0.31, 0.39)	<0.001
Multivariable[1]	1	0.97 (0.89, 1.06)	0.88 (0.80, 0.96)	0.83 (0.75, 0.91)	0.61 (0.54, 0.69)	<0.001

1. Adjusted for age, BMI, energy intake, smoking, family history of diabetes, oral contraceptive use, menopausal status and postmenopausal hormone use ("all women" analysis only), physical activity, alcohol intake, multivitamin use

Supplemental table 5: Hazard ratio for type 2 diabetes according to quintiles of Minimum Diet Diversity score for Women in the Nurses' Health Study II (*n*=88,520)

	**Q1**	**Q2**	**Q3**	**Q4**	**Q5**	***P* trend**
**All women**						
Median score	2.5	3.7	4.3	5.0	6.0	
No. of cases	1258	1348	1216	1260	1223	
Person years	345,160	367,749	386,592	368,701	365,161	
Age &kcal adjusted	1	0.94 (0.87, 1.02)	0.80 (0.74, 0.88)	0.79 (0.72, 0.87)	0.67 (0.61, 0.74)	<0.001
Multivariable[1]	1	1.08 (0.99, 1.17)	1.04 (0.95, 1.13)	1.11 (1.01, 1.21)	1.09 (0.99, 1.21)	0.88
**Women < age 50**						
Median score	2.3	3.5	4.0	5.0	6.0	
No. of cases	440	448	462	446	438	
Person years	179,652	196,562	227,925	201,628	191,986	
Age &kcal adjusted	1	0.94 (0.82, 1.08)	0.73 (0.63, 0.85)	0.76 (0.65, 0.88)	0.67 (0.57, 0.78)	<0.001
Multivariable[1]	1	1.10 (0.96, 1.26)	0.98 (0.84, 1.13)	1.09 (0.93, 1.27)	1.10 (0.93, 1.29)	0.64
**Women age ≥50**						
Median score	2.8	3.8	4.5	5.0	5.9	
No. of cases	818	868	754	814	785	
Person years	165,509	171,187	158,668	167,074	173,176	
Age &kcal adjusted	1	0.94 (0.85, 1.04)	0.85 (0.76, 0.95)	0.81 (0.72, 0.91)	0.68 (0.60, 0.77)	<0.001
Multivariable[1]	1	1.07 (0.97, 1.19)	1.08 (0.97, 1.21)	1.12 (1.00, 1.26)	1.10 (0.97, 1.24)	0.82

1. Adjusted for age, BMI, energy intake, smoking, family history of diabetes, oral contraceptive use, menopausal status and postmenopausal hormone use ("all women" analysis only), physical activity, alcohol intake, multivitamin use
